# Integrating antimicrobial stewardship into outpatient STI prevention and sexual health care

**DOI:** 10.1017/ash.2026.10883

**Published:** 2026-09-25

**Authors:** Bindu Mayi, Payal K Patel, Aniruddha Hazra

**Affiliations:** 1 Illinois College of Osteopathic Medicine, USA; 2 Intermountain Health Care Inc: Intermountain Healthcare, USA; 3 Section of Infectious Diseases and Global Health, University of Chicago Medicine, Chicago, IL, USA

## Abstract

Antimicrobial stewardship (AMS) is important in outpatient sexual health care because bacterial sexually transmitted infection (STI) management frequently involves recurrent antimicrobial exposure within select populations. Antimicrobial resistance (AMR) has narrowed treatment options and prompted repeated revisions to treatment guidelines, specifically for gonorrhea. Doxycycline postexposure prophylaxis and recurrent antibiotic exposure have intensified stewardship concerns in sexual health care. As outpatient AMS programs grow nationally, merging expertise on sexual health could optimize care and address AMR. We propose a practical operational framework for integrating AMS into outpatient sexual health care through clinical decision support, appropriate diagnostic testing and stewardship-informed antimicrobial prescribing, coupling prescribing practices with AMR surveillance, coordinated oversight between STI and stewardship programs, and patient-centered counseling. Stewardship integration must preserve timely access to care and trust within sexual health services. AMS integration may help sustain antimicrobial effectiveness, strengthen clinical decision-making, and support equitable, evidence-informed sexual health care amid evolving STI epidemiology and emerging AMR threats.

## Burden of sexually transmitted infections, novel biomedical prevention strategies, and the stewardship gap

Although overall reported rates of sexually transmitted infections (STIs) in the United States have recently shown signs of stabilization, certain populations continue to account for a disproportionate burden of reportable bacterial STIs. Adolescents and young adults (AYAs) ages 15–24 years, as well as men who have sex with men (MSM) and transgender women (TGW), remain disparately affected, with these inequities shaped by social and healthcare access factors, sexual network dynamics, confidentiality concerns, and uneven access to patient-centered sexual health services.^1–4^ Concurrently, antimicrobial resistance (AMR) has reshaped the treatment landscape for bacterial STIs. *Neisseria gonorrhoeae* has progressively acquired resistance to multiple antibiotic classes over decades, narrowing effective treatment options and prompting repeated modifications to treatment guidelines.^5,6^

Meanwhile, recent randomized controlled trials have demonstrated that doxycycline postexposure prophylaxis (doxy-PEP), 200 mg of doxycycline of any formulation taken within 72 hours after sex, substantially reduces incident chlamydia and syphilis and provides moderate protection against gonorrhea among MSM and TGW.^7–10^ As a result, both the Centers for Disease Control and Prevention (CDC) and World Health Organization (WHO) now support consideration of doxy-PEP for select populations as part of comprehensive sexual health services.^8,10^ However, expanding doxycycline exposure within populations introduces new antimicrobial stewardship (AMS) considerations related to long-term antimicrobial exposure, resistance surveillance, and stewardship-informed implementation of doxy-PEP within sexual health systems.

Emerging real-world and surveillance data suggest that doxy-PEP effectiveness against gonorrhea may vary according to local tetracycline resistance patterns in circulating *N. gonorrhoeae* strains, and that expanded doxycycline exposure may influence resistance patterns in bacteria other than the intended STI pathogens (off-target bacteria).^9,11^ At the same time, population-level reductions in chlamydia and syphilis incidence associated with doxy-PEP implementation may reduce downstream antibiotic utilization within all populations.^11–15^ Together, these considerations reinforce the importance of ongoing AMR surveillance, monitoring of prescribing practices, and stewardship-informed doxy-PEP implementation strategies.^9–11,16^

Despite their growing relevance to one another, AMS and STI services have generally developed within separate healthcare and public health infrastructures.^17,18^

## Why STI prevention and AMS remain structurally separate

Most STI diagnosis and treatment occur within outpatient clinics, urgent care centers, emergency departments, public health clinics, and community-based sexual health services.^4,5^ These settings frequently operate outside traditional hospital-based AMS infrastructures and often have limited dedicated stewardship infrastructure.^17^ However, outpatient antibiotic stewardship has expanded substantially through national initiatives led by organizations such as the CDC and The Joint Commission.^17^ Although antimicrobial allergy assessment and de-labeling are also recognized components of outpatient AMS, this commentary focuses specifically on opportunities to operationally integrate stewardship activities within outpatient STI prevention and sexual health care. Early outpatient stewardship efforts often concentrate on inappropriate prescribing in acute respiratory infections but integrating this growing field with STI-related antibiotic prescribing could certainly yield public health and health-system wins.^17^ STI-related antibiotic prescribing is not routinely incorporated into formal AMS dashboards or quality review structures in many ambulatory settings, despite representing an important and often underrecognized source of cumulative antimicrobial exposure.^17,19,20^ One large health system used antibiotic stewardship resources to improve HIV and STI co-testing, thereby increasing testing in urgent care and emergency department settings and improving linkage to care, illustrating how existing stewardship infrastructure can support broader sexual health quality improvement initiatives.^21^ Integrating outpatient stewardship efforts with sexual health care presents several opportunities to optimize antibiotic use, including treatment for recurrent STIs in the setting of ongoing exposure within high-incidence sexual networks, expedited partner therapy, STI retesting to detect repeat infections, and stewardship-informed implementation of doxy-PEP in recommended populations. These proposed stewardship opportunities are intended to inform future implementation and evaluation of integrated outpatient AMS within STI prevention and sexual health care. Recurrent antimicrobial exposure across these clinical contexts may contribute to cumulative antimicrobial selection pressure at the population level, particularly among AYAs, who experience disproportionately high STI incidence and repeat infections, even when individual prescriptions remain guideline-concordant.^5,19,22^ These considerations further underscore the importance of integrating AMR surveillance, prescribing review, and stewardship-informed implementation within outpatient sexual health systems.^7–11,16^

## Operational integration of AMS into STI prevention and care

Embedding AMS into outpatient STI prevention and care does not require creation of parallel stewardship systems. Integration could occur through operational tools embedded within existing sexual health workflows, including standardized electronic medical record (EMR) order sets, clinical decision-support tools, prescribing guidance linked to current AMR surveillance data, and integration of STI-related prescribing metrics into ambulatory stewardship review processes.^23^ At a health system level, these approaches could improve laboratory, diagnostic, and antimicrobial stewardship support guideline-concordant prescribing and improve long-term patient outcomes for vulnerable populations.^5,17,20^ Health system AMS programs are more likely to have access to data analysts (often experienced in antibiotic use data), information technology support, and EMR resources. They also have expertise in analyzing prescribing data, providing feedback to clinicians, and implementing system-level improvements to optimize antimicrobial use.

**Figure 1.**
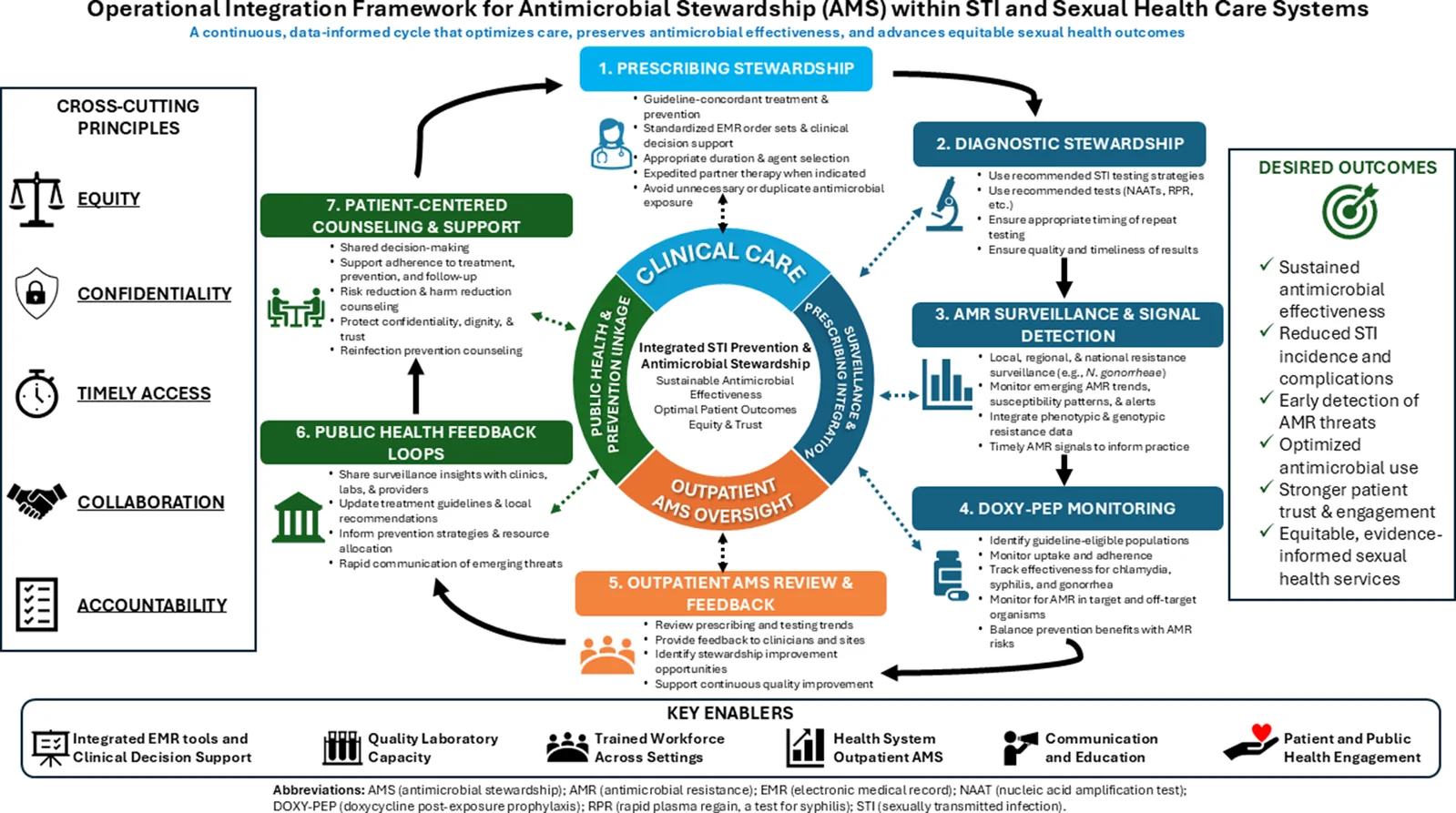
Figure 1 long description. Operational integration framework for antimicrobial stewardship (AMS) within outpatient sexually transmitted infection (STI) and sexual health care systems.

Importantly, AMS-informed STI care must preserve timely access to diagnosis and treatment, confidentiality, and trust. If implemented without attention to these priorities, stewardship initiatives could unintentionally introduce additional barriers to care by increasing treatment delays, administrative complexity, or concerns regarding confidentiality and stigma. Barriers to accessing sexual health services, including stigma, confidentiality concerns, cost, fragmented access to follow-up care, and limited availability of youth-centered services, may delay diagnosis and treatment, contribute to recurrent infection, and increase cumulative antimicrobial exposure.^3,24^ Delays in treatment while awaiting laboratory confirmation, referral, follow-up appointments, or partner evaluation may increase ongoing transmission and contribute to adverse reproductive health outcomes.^5^ Stewardship integration should therefore emphasize clear communication with patients regarding antimicrobial use, reinfection prevention, and AMR while preserving affirming, patient-centered sexual health care.^3,4,24^ Youth-centered counseling should remain confidential, developmentally appropriate, and focused on helping AYAs understand treatment adherence, partner management, reinfection prevention, and the importance of preserving long-term antimicrobial effectiveness.^3–5^ Figure 1 summarizes a proposed operational integration framework for embedding stewardship principles within outpatient STI prevention and sexual health systems. Surveillance and Prescribing **I**ntegration. The WHO’s Integrated Drug Resistance Action Framework emphasizes surveillance, governance, and stewardship across infectious disease threats, including stronger linkage between laboratory systems, resistance monitoring, prescribing practices, and public health response strategies.^25^ Within STI prevention systems, more systematic integration of antimicrobial prescribing data with AMR surveillance systems may strengthen AMS and improve early recognition of emerging resistance patterns. Operationally, stewardship integration could link several complementary data streams. These include antimicrobial prescribing and dispensing data from electronic health records, outpatient prescribing records, health-system pharmacies, or administrative claims; doxy-PEP utilization captured through prescriptions or pharmacy fills; repeat infection patterns identified through STI testing records or public health surveillance registries; and local susceptibility data from the CDC’s Combatting Antimicrobial Resistant Gonorrhea and Other STIs (CARGOS) project, public health laboratories, or regional laboratory surveillance programs, including phenotypic and genotypic resistance data where available.^5,26,27^

Linking and reviewing these data can be facilitated by state health departments. In the last few years, almost all U.S. state health departments have hired at least one full-time antimicrobial stewardship lead to champion antibiotic stewardship.^28,29^ A feasible model would involve periodic review by local or state antibiotic stewardship and STI program leadership in collaboration with public health epidemiologists, laboratory partners, STI clinical leads, and regional stewardship leaders. Coupling prescribing trends, doxy-PEP utilization, repeat infection patterns, and local susceptibility data may help identify evolving AMR pressures within high-incidence populations before widespread treatment failures become clinically apparent.^9,25^Even partial integration between prescribing records and surveillance systems may support stewardship-informed STI prevention. For example, programs could begin with quarterly review of aggregate STI-related antimicrobial prescribing volume, doxy-PEP utilization, repeat STI diagnoses, and local gonococcal susceptibility alerts without requiring full patient-level data integration. Importantly, stewardship evaluation of doxy-PEP should balance potential AMR risks with the substantial prevention benefits associated with reduced chlamydia and syphilis incidence, including the potential for reduced cumulative antibiotic exposure among doxy-PEP users and, through reduced STI transmission and fewer subsequent infections requiring treatment, among non-users.^12–15^

These integrated surveillance approaches may help programs identify emerging resistance signals, refine prescribing guidance, support evidence-informed doxy-PEP implementation, update provider education, and align prevention strategies with evolving local epidemiology while minimizing unnecessary antimicrobial exposure.^9,15^ One of the greatest barriers to stewardship-informed STI care remains limited outpatient diagnostic capacity for detection of AMR. This challenge is particularly important for sexually transmitted pathogens such as *N. gonorrhoeae* and *Mycoplasma genitalium*, where evolving resistance patterns increasingly complicate empiric treatment selection and may require more individualized therapeutic approaches. Development of molecular diagnostics capable of identifying STI pathogens and clinically relevant resistance determinants may substantially strengthen individualized treatment selection, reduce unnecessary empiric antibiotic exposure, and improve resistance surveillance within outpatient sexual health settings.^5,9,25^ However, broader implementation will require improved diagnostic availability, reduced costs, and continued validation of clinically relevant resistance markers before these technologies can be more widely adopted across outpatient sexual health settings.

## Shared oversight and governance

Translating stewardship integration into clinical practice requires coordination between STI programs and AMS governance structures. Public health agencies already issue treatment advisories and guideline updates in response to emerging epidemiologic and resistance signals, including gonorrhea treatment recommendations and doxy-PEP guidance informed by CARGOS.^5,25^ Incorporating review of STI-related antimicrobial prescribing patterns within existing ambulatory stewardship activities, public health STI surveillance and review programs, and health-system stewardship programs may help ensure that resistance trends, prescribing practices, and clinical workflows are reviewed together rather than independently.^11,30,31^ Such coordination may also facilitate more rapid communication of emerging resistance threats, evolving susceptibility patterns, and treatment guidance updates across clinical and public health systems.^5,25–27,30,31^

Framing AMS solely as a restriction on antimicrobial use may risk undermining trust within sexual health services if stewardship efforts are perceived as barriers to timely, confidential, or nonjudgmental care. Reduced trust may discourage care-seeking, testing, disclosure of sexual behaviors, partner notification, or follow-up engagement, potentially contributing to ongoing transmission and delayed treatment within high-incidence populations.^3,4,24^ In contrast, integrating AMS into outpatient STI prevention and sexual health care can strengthen clinical care by supporting appropriate diagnostic testing, individualized risk assessment, evidence-informed antimicrobial prescribing, and preservation of treatment effectiveness.^4,5,11^ These complementary roles illustrate that STI prevention and AMS should be viewed as mutually reinforcing rather than competing public health objectives.

## AYAs as a strategic target population for stewardship integration

Although stewardship integration is relevant across outpatient STI care settings, AYAs remain an important population within STI prevention because of persistently high STI incidence, recurrent treatment exposure, and barriers to longitudinal sexual health care engagement.^1,3,5^ Stewardship efforts targeting AYAs therefore require approaches that support repeated episodes of STI prevention, diagnosis, treatment, and follow-up while minimizing unnecessary antimicrobial exposure over time. These efforts should preserve confidentiality, timely access, and nonjudgmental counseling while supporting appropriate antimicrobial use and patient education regarding treatment adherence, partner management, and reinfection prevention. Provider education may be particularly important in youth-serving settings, where familiarity with doxy-PEP and comfort with prescribing remain variable among pediatric providers.^32^

## Conclusion

The convergence of evolving AMR, recurrent antimicrobial exposure, and expanding outpatient stewardship creates an opportunity for operational integration of AMS into outpatient STI prevention and sexual health care. Existing outpatient sexual health infrastructures provide a practical foundation for linking clinical workflows, antimicrobial prescribing, AMR surveillance, and coordinated stewardship oversight while preserving timely, equitable, and patient-centered sexual health services. As outpatient stewardship programs continue to expand, integrating stewardship principles into outpatient STI prevention and sexual health care may help sustain antimicrobial effectiveness while strengthening evidence-informed, equitable STI prevention and sexual health care.

The framework illustrates a continuous, data-informed operational cycle linking clinical care, surveillance and prescribing integration, stewardship oversight, and public health and prevention linkage within outpatient STI and sexual health care systems. The surrounding stewardship domains represent the major operational activities through which AMS is integrated into outpatient STI and sexual health care, including prescribing stewardship, diagnostic stewardship, AMR surveillance and signal detection, doxy-PEP monitoring, stewardship review and feedback, public health feedback loops, and patient-centered counseling and support. The cross-cutting principles guiding AMS integration include equity, confidentiality, timely access to care, collaboration, and accountability. The key enabling infrastructure components include integrated EMR tools and clinical decision support; quality laboratory capacity; workforce training across outpatient, laboratory, and public health settings; integration of prescribing, laboratory, and surveillance data; communication of evolving AMR trends and updated treatment guidance, as well as provider education; and patient and public health engagement. Dashed bidirectional arrows indicate ongoing coordination and exchange of information between the central stewardship domains illustrated in the circle and the surrounding stewardship activities within outpatient STI and sexual health care systems. This framework illustrates a proposed operational model for integrating AMS principles into outpatient STI prevention and sexual health care.

Operational integration framework illustrating AMS within outpatient sexually transmitted infection (STI) and sexual health care systems. A circular operational cycle links four core domains: clinical care, surveillance and prescribing integration, stewardship oversight, and public health and prevention linkage. Surrounding the cycle are seven implementation components: prescribing stewardship, diagnostic stewardship, AMR surveillance and signal detection, doxy-PEP monitoring, stewardship review and feedback, public health feedback loops, and patient-centered counseling and support. Cross-cutting principles include equity, confidentiality, timely access to care, collaboration, and accountability. Key enabling infrastructure includes integrated electronic medical record tools and clinical decision support, quality laboratory capacity, workforce training across outpatient, laboratory, and public health settings, integration of prescribing, laboratory, and surveillance data, communication of evolving AMR trends and updated treatment guidance, provider education, as well as patient and public health engagement. Desired outcomes include sustained antimicrobial effectiveness, reduced STI incidence and complications, early detection of AMR threats, optimized antimicrobial use, stronger patient trust and engagement, and equitable, evidence-informed sexual health services.

## Figure 1. Long description

A diagram of the operational integration framework for antimicrobial stewardship (AMS) within STI and sexual health care systems. The framework is divided into several key components: Prescribing Stewardship, Diagnostic Stewardship, AMR Surveillance & Signal Detection, Doxy-PEP Monitoring, Outpatient AMS Review & Feedback, Public Health Feedback Loops, and Patient-Centered Counseling & Support. These components are interconnected and form a continuous cycle aimed at optimizing care, preserving antimicrobial effectiveness, and advancing equitable sexual health outcomes. The diagram also highlights cross-cutting principles such as equity, confidentiality, timely access, collaboration, and accountability. Key enablers include integrated EMR tools and clinical decision support, quality laboratory capacity, trained workforce across settings, health system outpatient AMS, communication and education, and patient and public health engagement. Desired outcomes include sustained antimicrobial effectiveness, reduced STI incidence and complications, early detection of AMR threats, optimized antimicrobial use, stronger patient trust and engagement, and equitable, evidence-informed sexual health services.

Navigate back to Figure 1..
